# Relationship between visual ability assessment and punch performance in competition in male amateur boxers

**DOI:** 10.3389/fphys.2024.1429554

**Published:** 2024-07-16

**Authors:** Rui Wu, Qingyuan Yang, Weijia Cui, Dongxu Gao, Yifei Luo, Dexin Wang

**Affiliations:** ^1^ School of Athletic Performance, Shanghai University of Sport, Shanghai, China; ^2^ Shanghai Key Lab of Human Performance, Shanghai University of Sport, Shanghai, China; ^3^ Xinjiang Research Institute of Sports Science, Urumqi, China

**Keywords:** sport visual ability, visual testing, vision training, combat sports, boxing, punch performance

## Abstract

Sport visual ability plays an important role in the performance of elite athletes in competition. However, its relationship with boxers’ performance has not been fully understood. This study investigated the relationship between sports visual abilities and in-competition punching performance in 26 highly trained male amateur boxers. Ten visual abilities of the boxers were tested using the Senaptec Sensory Station (Senaptec, Beaverton, OR, United States), including Visual Clarity (VC), Contrast Sensitivity (CS), Depth Perception (DP), Near Far Quickness (NFQ), Target Capture (TC), Perception Span (PS), Multiple Object Tracking (MOT), Eye-Hand Coordination (EHC), Go/No Go (GNG), and Reaction Time (RT). Performance analyses were conducted on national boxing championships conducted by the boxers to analyze their punching accuracy. Correlation and regression analyses showed that punch performance %Hit was very strong correlated with DP, EHC, GNG, and RT, and showed a strong correlation with VC, CS, and PS. %Hit was moderate correlated with MOT, while there was no correlation with NFQ and TC. In addition, RT, EHC, and DP are important visual ability variables for boxers. The results of this study indicate that there is an overall relationship between sports visual ability and boxing performance, but there is also a specific relationship between variables in visual ability and boxing performance, especially faster reaction times, better processing of visual information, and decision-making abilities, and the ability to accurately recognize the distance and position of an opponent and their punches are essential for enhancing boxing performance. Further studies are needed to investigate the relationship between sport visual ability and more comprehensive performance in boxers, and the possibility of enhancing performance through specific visual training.

## 1 Introduction

Boxing is an ancient sport with a long Olympic history and a solid professional base worldwide (Bianco et al., 2013). Boxing performance depends on several factors such as physical, technical, tactical, and cognitive abilities (Bianco et al., 2011; Darby et al., 2014; Loturco et al., 2021). For boxers to achieve elite competitive levels, they need to develop a variety of performance factors, such as optimal levels of strength and explosive power, as well as aerobic and anaerobic capacity (Chaabène et al., 2015). Several studies have shown that strength is a key factor in the outcome and success of a boxing match (Bianco et al., 2013; Lenetsky et al., 2013; Chaabène et al., 2015; López-Laval et al., 2020). Some studies have illustrated that speed and acceleration are the best variables to determine a boxer’s punch performance (Loturco et al., 2014; Loturco et al., 2018). In addition, there is a great deal of high-intensity action in the three rounds of an amateur boxing match, and its short breaks are not enough to allow the athlete to fully recover (El-Ashker and Nasr, 2012). Therefore, the contribution of anaerobic and aerobic capacity to boxing performance is very important (Arseneau et al., 2011; Bruzas et al., 2014). In boxing, athletes are required to perform a large number of offensive and defensive actions (Davis et al., 2013; Slimani et al., 2017), and in addition, experienced boxers can punch for around 402–405 milliseconds (Stanley et al., 2018; Menzel and Potthast, 2021). This requires the boxer to concentrate and to see the opponent through their eyes. Perceptual-cognitive abilities include perception, attention, anticipation, inhibitory control, and cognitive flexibility, which may determine whether a boxing match is won or lost (Stiller et al., 2014). Thus, perceptual-cognitive abilities, including visual abilities, contribute significantly to boxing performance. Sport visual ability also seems to be an important element for boxers.

Sports vision is an emerging field that seeks to establish the relationship between visual ability (or visual skills) and sports performance (Laby and Appelbaum, 2021). Approximately 80% of the information that athletes acquire about their sports environment is through their visual abilities and their components (Buys and Ferreira, 2008). In addition, approximately 70% of the body’s sensory receptors are located in the eye (Ramaja and Hansraj, 2022). Several studies have reported that vision is the key to success in almost all sports (Erickson, 2021; Laby and Appelbaum, 2021). In addition, vision and visual processing are recognized as important components of successful athletic performance (Erickson, 2018; Erickson, 2021). Various sports rely on vision to guide precise and difficult movement actions, and this union of perception and action has been identified as a key element limiting excellence in various sports (Le Runigo et al., 2005). Also, different sports require different visual abilities (Nascimento et al., 2020). In addition, vision is the basis for coaches and athletes to explore methods that can enhance athletic performance (Paul et al., 2011).

There has been a great deal of research showing that sports visual ability is associated with better performance in athletic competition and in some cases a reduction in sports injuries, with most of this research focusing on baseball (Reichow et al., 2011; Burris et al., 2018; Laby et al., 2018; Laby et al., 2019; Liu et al., 2020), and other sports including field hockey (Poltavski and Biberdorf, 2015), ice hockey (Martell and Vickers, 2004), rugby (Ludeke and Ferreira, 2004; Millard et al., 2021), basketball (Mangine et al., 2014), and shooting (Causer et al., 2010). And none have been found in combat sports. These studies support the idea that there is a definite link between improving visual ability and enhancing athletic performance. One of the concepts of sport vision training is that aspects of visual ability are important for a given sport (Blundell, 1985; Abernethy, 1986; Ciuffreda et al., 2004; Laby and Appelbaum, 2021). Sports visual abilities generally include Visual Clarity, Contrast Sensitivity, Depth Perception, Perception span, Objective Tracking, Target Capture, Near Far Quickness, Eye-Hand Coordination, Go/No Go, and Reaction Time (Regan and Gray, 2001; Erickson et al., 2011; Wang et al., 2015), and these abilities have been identified as important for sports performance (Boff et al., 1986; Hitzeman and Beckerman, 1993; Elmurr, 2011; Erickson, 2012; Ellison, 2015; Erickson, 2021; Huppert et al., 2021; Erickson, 2022). Moreover, athletes have better visual abilities than non-athletes, and good athletes have better visual abilities than poorer athletes (Stine et al., 1982).

Since the first modern Olympics in 1904, the rules of competition in boxing have changed many times (Davis, 2012; Bianco et al., 2013). In 2013, the Amateur International Boxing Association (AIBA) made a major change to the rules of amateur boxing, with the refereeing system being changed to a “Ten Point Must-System.” In the “Ten Point Must-System,” the judges base their decisions on four criteria: the number of quality punches on the target area, technical and tactical superiority in dominating the bout, competitiveness, and infringement of the rules (AIBA, 2021). Of these, the number of high-quality punches to the target area is the most critical criterion for awarding penalties. Davis et al., (2018) and Emily et al. identified punch accuracy (percentage of hits) as an important criterion that is considered a major sign of dominance in boxing (Dunn et al., 2017). As a result, boxers strive to be able to have a high punching accuracy as a way to enhance their punching performance in the match. In boxing, boxers use various techniques to hit their opponents while avoiding their punches (Guidetti et al., 2002). They need to have quick reflexes and be quick to throw punches (Bianco et al., 2008; VencesBrito et al., 2011; Darby et al., 2014). In this process, vision plays a key role in providing the information needed to react accurately and quickly (Martínez de Quel and Bennett, 2019).

Of the current studies on boxing related to sports vision, Badau et al. reported that boxers had the best reaction times (Badau et al., 2018). Laby et al. compared the visual abilities of Olympic athletes and noted that boxers need more integrated visual abilities, with reaction time and eye-hand coordination being the most important (Laby et al., 2011). These are the only studies that have included visual abilities in boxing, but these reports are not comprehensive. To the authors’ knowledge, no previous research has been found on the relationship between boxing and sports visual abilities. Therefore, the main purpose of the present research was to examine the relationship between amateur boxers’ punching performance and sports visual abilities in match play. The aim is to set a starting point for future research on the integration of sport vision with combat sports (e.g., boxing) and to provide possible options for performance improvement in boxers. The hypothesis of this study is that some variables in sports visual ability are positively correlated with amateur boxers’ punching performance.

## 2 Materials and methods

### 2.1 Participants

Twenty-six highly trained elite male amateur boxers (age: 20.65 ± 2.8; height: 183.65 ± 5.79; weight: 78.78 ± 11.92; experience: 8.19 ± 2.8) participated in this study. All boxers are within the margins permitted by the rules of boxing competition. Their weight classifications are also within the range of weight classifications required by the Technical and Competition Rules of Amateur Boxing, i.e., Flyweight to Super Heavyweight (51 KG-92 + KG). Inclusion criteria were as follows: a) at least 5 years of boxing-specific training as a professional boxer; b) participation in at least 5 national and 3 international tournaments; c) at least top three finishes in national tournaments; d) subjects had normal visual acuity and visual functioning, with no ocular diseases or corrective treatments; and e) no prior training in visual abilities. All participants were fully informed about the aims, benefits, and potential risks of the study. The study procedures were approved by the Ethics Committee of the Shanghai University of Sport (Ethics number: 102772023RT130) and complied with the standards set out in the latest version of the Declaration of Helsinki (Association, 2013). All participants signed a written informed consent form.

### 2.2 Materials

#### 2.2.1 Sport visual ability tests

The Senaptec Sensory Station (Senaptec, Beaverton, OR, United States) was used to assess 10 visual abilities with demonstrated reliability [for a detailed description of the reliability and validity of the program, see Erickison et al., (2011), Wang et al., (2015), Poltavski and Biberdorf, 2015, Knöllner et al., (2022), and Appelbaum and Erickson, (2018)]. The Senaptec Sensory Station consists of a battery of ten computerized visual sensorimotor tasks, each designed to evaluate a specific aspect of a participant’s visual-motor abilities. The measures of each visual ability are as follows:

The *Visual Clarity (VC)* task assessed the minimum detectable spatial resolution for a non-moving object. In this task, a black Landolt ring with a gap appears on a white background in the center of the screen. The gap can be at the top, bottom, left, or right side of the ring. Participants stood 3 m away from the screen and their task was to swipe the smartphone screen in the direction of the gap.

The *Contrast Sensitivity (CS)* task assessed the minimum level of contrast to distinguish light from dark. Four black circles were displayed in a diamond-shaped structure on a light gray background on the screen, one of which would randomly contain a pattern of concentric rings of varying brightness. Participants stood 3 m away from the screen and their task was to swipe in the direction of the circle with the pattern on their smartphone.

The *Depth Perception (DP)* task assessed the minimum amount of difference required to discriminate depth differences. On the screen, four black circles were arranged in a diamond-shaped structure. Participants stood 3 m from the screen and wore a pair of red/blue glasses. Participants were asked to swipe in the direction of the ring that appeared to have depth on their smartphone.

The *Near Far Quickness (NFQ)* assesses the speed and accuracy of adapting to near and far targets, requiring a rapid adaptation-vergence response. Subjects were 3 m from the screen, holding a smartphone, and 40 cm away. During the test, a black Landolt ring was alternately presented at the center of the “far” of the screen and the top of the “near” of the smartphone screen. The size of the Landolt ring was adjusted according to the individual’s visual acuity. The task was to swipe the smartphone screen as fast as possible to indicate the opening of the Landolt ring within 30 s. The score is the total number of correct responses within 30 s.

The *Target Capture (TC)* task assessed the ability to resolve a brief target presented in the periphery. A black Landolt ring has randomly appeared on one of the four corners of the screen. The presentation duration of the Landolt ring started at 250 ms and was dynamically adjusted as it progressed. Participants were tasked with identifying the direction of the gap on the Landolt ring by swiping the smartphone screen. The measurement distance was 3 m. The final threshold is the minimum amount of presentation time (in milliseconds) required to correctly identify the gap in the ring.

The *Perception Span (PS)* task assesses the ability to identify the spatial workings of a briefly presented pattern of dots. In this task, a radial network of white circles was displayed on the touchable screen. At the time of measurement, a subset of the circle was briefly filled with black dots, and participants were required to reproduce the pattern of flashing black dots by touching the corresponding circle. There are a total of 11 levels of tasks that participants may complete, and the spatial pattern of black dots becomes more complex as the size of the grid increases at each level. The task ended when the participants could no longer achieve a passing score on two successive trials for a given level. Participants stood an arm’s length away from the screen. The PS score is the total number of correctly identified dots minus the number of missed or falsely identified dots.

The *Multiple Object Tracking (MOT)* task assessed the ability to accurately track multiple moving objects at different speeds. In this task, several sets of dots appear on the screen, each consisting of a red dot and a black dot. During the test, the red dots will turn black, and then all groups of dots will start to rotate. Participants need to track the dot that starts red and tap it out when it stops rotating. As the task level increases, the number of groups of dots increases, and the speed of rotation also increases. The task ended when the participants made two consecutive errors on a given level. Participants stood an arm’s length away from the screen.

The *Eye-Hand Coordination (EHC)* assesses the ability to process and respond to visual stimuli. A grid of 80 (10 columns × 8 rows) equally spaced black circles was displayed on the screen. During the test, one of the circles will turn green. Participants were asked to touch the dot as rapidly as possible with either hand, and it would then immediately appear in a new location. Subjects were required to successfully complete a sequence of 80 point presentations and the total time required to complete the sequence was measured. Participants stood an arm’s length away from the screen.

The *Go/No Go (GNG)* task tested the ability to respond quickly to “go” targets while inhibiting responses to “no-go” non-targets. This task is similar to EHC, but the dots were presented for only 500 ms and they could be either red or green. Participants were tasked with touching the green dot as fast as possible, but not responding to the red dot. The GNG score is the total number of green dots successfully touched minus the total number of red dots incorrectly touched.

The *Reaction Time (RT)* task measured simple motor reaction time in response to a visual stimulus. In this task, two rings appear on either side of the touch screen. Participants were asked to place the fingers of each hand on each of the two rings and to turn green when placed on the rings. Once one of the green rings turned red, the subject quickly disengaged it and then replaced it. Reaction time was calculated as the average reaction time of all subjects completing all measurements.

#### 2.2.2 Boxing performance analysis

A video camera (Sony FDR-AX700, CHN) was used to record each boxer’s bouts during the National Boxing Championships in which all boxers participated. The boxers were analyzed in high-definition for each bout in which they participated and their on-field performance data was recorded. It is analyzed by a qualified boxing performance analyst through slow-motion replay, which is adjusted in 0.1-s increments for accurate viewing. All matches were analyzed by the same analyst and five matches were randomly analyzed twice to check for internal consistency (ICC). In addition, the same five bouts were analyzed by a second experienced analyst to determine the intertest reliability of the primary analyst. A detailed record of the number of punches thrown by a boxer in a round or bout, the number of punches that successfully hit the target, and the number of punches that missed the target (Table 1). The performance indicator chosen for this study was Punch accuracy (calculated based on the number of punches that hit the target as a percentage of the total number of punches landed. Unit: %Hit). For more details on performance indicator parameters and boxing performance analysis, see the studies by Thomson et al., (2013), (Davis et al., 2013; Davis et al., 2015; Davis et al., 2016; Davis et al., 2018), and Emily et al. (Dunn et al., 2017).

**TABLE 1:** Description of boxing performance analysis variables.

Variable	Unit	Description
Punches Thrown	Number	Total number of punches thrown
Hit	Number	A punch that hits the target area
Miss	Number	A punch that misses the target area
%Hit	%	Number of hits to the target area as a percentage of total punches thrown

### 2.3 Procedures

The present study consisted of two assessments. Firstly, the 10 visual ability test tasks based on the Sports Vision Station were conducted 1 week before the competition period, and all the test tasks were completed in 1 day again. The second is the analysis of the in-field punching performance of the boxers performed during the competitive period.

The sport visual ability test is administered in the SPORTS VISUAL PERFORMANCE LABORATORY. All boxers complete a visual ability assessment within 1 week before participating in the National Boxing Championships. Subjects arrived at the SPORTS VISUAL PERFORMANCE LABORATORY and informed consent was obtained from each individual, after which descriptive questions about the participant were registered, including name, age, gender, height, and hand/foot preference. In addition, their sports category and sports information (level, primary/secondary sport, location), vision correction, and concussion history were included. The boxer then completes an approximately 25-min sports visual ability assessment. Visual ability was assessed as ten computerized visual sensorimotor tasks, each of which was first shown an animation example and then practiced in 3 trials, followed by a formal testing session. If the participants had difficulty recognizing it, they were encouraged to guess based on the instructions.

Data acquisition for the boxing performance analysis took place during the season 2023/2024. A video camera was used to record the matches of each boxer during the National Boxing Championships in which all boxers participated during the 2023/2024 season. Analyzed by a qualified boxing performance analyst. This analyst is qualified as a boxing referee and has extensive experience analyzing match performance. Conferences were held with elite boxing coaches before conducting the boxing match analysis as a means of developing the variables (punches, hits, and misses) to be recorded during the analysis.

### 2.4 Statistical analysis

Data were analyzed using IBM SPSS Statistics (version 27.0) and expressed as mean ± SD. The normality of the data was tested using the Shapiro-Wilk test. Pearson’s correlation coefficients (r) and their corresponding 95% confidence intervals (95% CI) were calculated to assess the linear relationship between visual ability and punching performance. Multiple regression analysis was used to assess the relationship between visual ability variables and punching accuracy in boxers. Assumptions of normality, linearity, homoscedasticity, and independence of the regression model were confirmed by distribution and residual analysis. The consistency of the results of the two analyses of five randomly selected matches, and the consistency between analysts, was checked by the interclass correlation coefficient (ICC). The interpretation was based on Cohen’s correlation coefficient r (Cohen, 1977): r = 0 is none, 0 to 0.3 is weak, 0.3 to 0.5 is moderate, 0.5 to 0.7 is strong, and 0.7 to 1 is very strong. The adjusted coefficient of determination (R^2^) was used to assess the goodness of model fit. The significance level was set at *p* < 0.05. Using G*power 3.1 a *post hoc* analysis of the statistical power of the model based on the observed effect size (Cohen’s ƒ^2^) and α = 0.05 was performed to determine the sensitivity of the model to Type II errors (Faul et al., 2009).

## 3 Results

All boxers completed the test tasks and bouts. The mean ICC value used to check the consistency of the results of the two analyses over the five bouts was 0.994. In addition, the inter-tester consistency ICC value used to check the primary analysts was 0.915. Both were within acceptable limits. Table 2 presents the descriptive statistics. Table 3 shows the correlation coefficients (Pearson’s r) and their 95% confidence intervals (95% CI) between boxers’ punching performance and visual ability parameters. Table 4 shows the results of the multiple stepwise regression analysis.

**TABLE 2:** Descriptive analysis of anthropometrics, visual ability tests, and punch accuracy.^a^

Parameter	M ± SD n = 26
Personal data	
Age(y)	20.65 ± 2.8
Height (cm)	183.65 ± 5.79
Weight (kg)	78.78 ± 11.92
Experience(y)	8.19 ± 2.8
Visual ability variables	
VC(logMAR−)	−0.08 ± 0.08
CS(logCS+)	1.75 ± 0.27
DP (arcsec−)	151.54 ± 51.73
NFQ (score+)	15.85 ± 4.27
TC (ms−)	294.23 ± 66.82
PS(score+)	42.85 ± 6.92
MOT (score+)	0.78 ± 0.11
EHC(s-)	51.57 ± 3.95
GNG (score+)	5.5 ± 1.14
RT (ms−)	302.46 ± 29.95
Punch variables	
Punch accuracy (%Hit)	26.28 ± 6.49

^a^ VC, visual clarity; CS, contrast sensitivity; DP, depth perception; NFQ, near far quickness; TC, target capture; PS, perception span; MOT, multiple object tracking; EHC, Eye-Hand Coordination; GNG, Go/No Go; RT, reaction time; + = higher is better; − = lower is better.

**TABLE 3:** Correlation coefficients between boxers’ visual ability and punching performance.^a^ ^b^

	VC	CS	DP	NFQ	TC
%Hit	−0.683 (−0.847 to −0.402)**	0.543 (0.197 to 0.769)**	−0.82 (−0.916 to −0.634)**	0.235 (−0.168 to 0.57)	−0.325 (−0.633 to 0.071)
	PS	MOT	EHC	GNG	RT
%Hit	0.501 (0.141 to 0.744)**	0.389 (0.002 to 0.675)*	−0.828 (−0.92 to −0.64)**	0.801 (0.6 to 0.907)**	−0.916 (−0.962 to −0.82)**

^a^ %Hit = hit rate of punches thrown.

^b^ Correlation coefficients and CI, 95% are shown.

*p < 0.05.

**p < 0.01.

**TABLE 4:** Standardized regression to explain the punch performance (%Hit).^a^

	β^a^	CI for β	*p*
RT	−0.441	−0.141 to −0.05	<0.001^**^
EHC	−0.346	−0.839 to −0.297	0.001^**^
DP	−0.297	−0.059 to −0.016	0.002^*^
Model fit	Adjusted R^2^ = 0.925		0.001^**^

^a^ %Hit = hit rate of punches thrown; RT, reaction time; EHC, Eye-Hand Coordination; DP, depth perception; βa = estimated standardized regression coefficient.

*p < 0.05.

**p < 0.01.

Correlation analyses revealed that of all the visual ability variables, punch performance %Hit displays a strong correlation with VC (r = −0.683, *p* < 0.001) and CS (r = 0.543, *p* = 0.004). %Hit showed a very strong correlation with DP (r = −0.82, *p* < 0.001) and a strong correlation with PS (r = 0.501, *p* = 0.009). %Hit showed a moderate correlation with MOT (r = 0.389, *p* = 0.049). There were very strong correlations with EHC (r = −0.828, *p* < 0.001), GNG (r = −0.828, *p* < 0.001), and RT (r = −0.916, *p* < 0.001). Whereas, NFQ and TC did not correlate with punch performance %Hit (Table 3).

**FIGURE 1:**
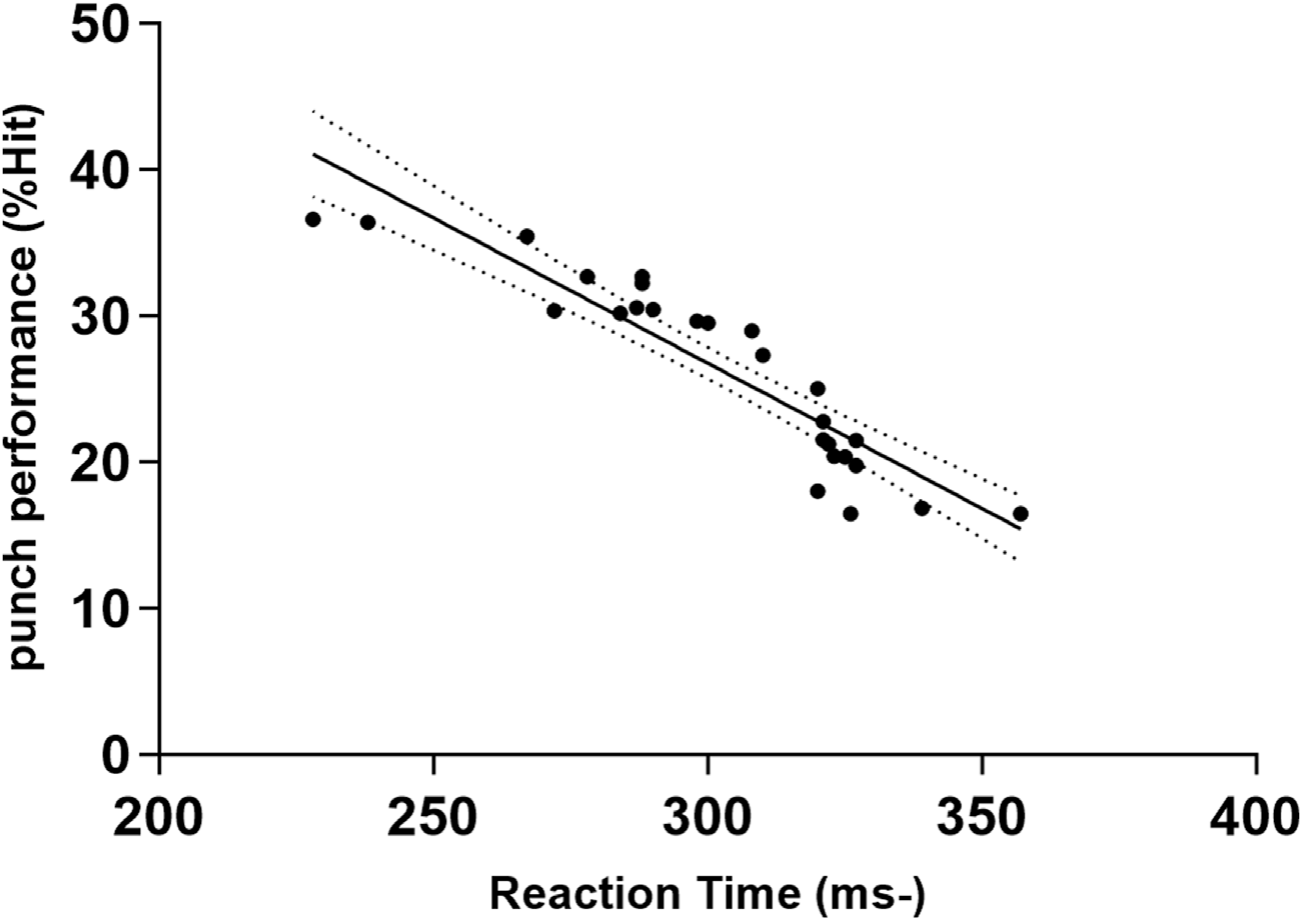
Bivariate correlation between punch performance and variables entered into the regression model: the Reaction Time (ms). The broken line represents 95% CI. −: lower is better.

**FIGURE 2:**
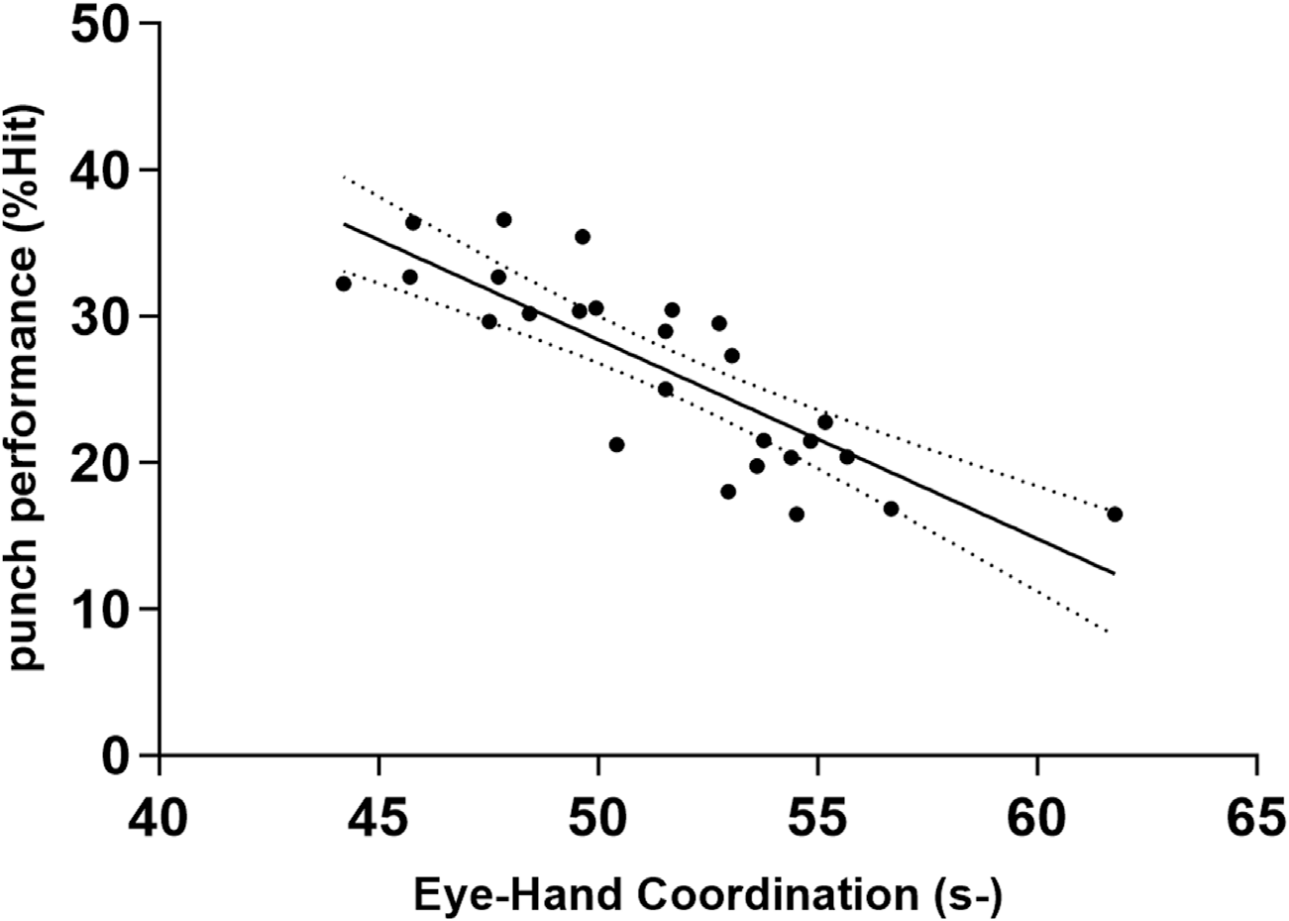
Bivariate correlation between punch performance and variables entered into the regression model: the Eye-Hand Coordination (s). The broken line represents 95% CI. −: lower is better.

**FIGURE 3:**
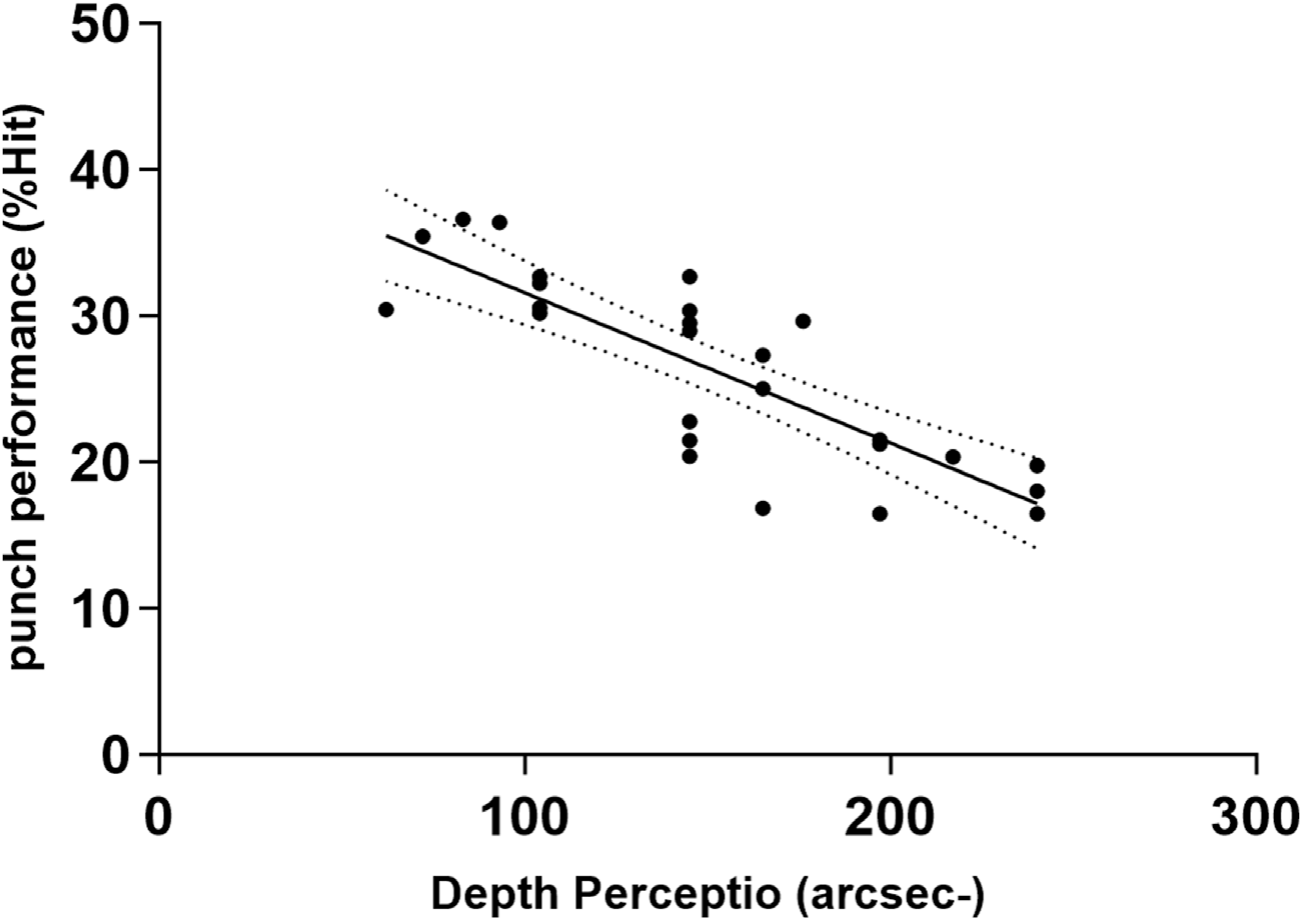
Bivariate correlation between punch performance and variables entered into the regression model: the Depth Perception (arcsec). The broken line represents 95% CI. −: lower is better.

Multiple stepwise regression confirmed that among all visual abilities, Reaction Time, Eye-Hand Coordination, and Depth Perception explained boxers’ punching performance. The goodness of fit was Adjusted R^2^ = 0.925 (*p* < 0.001). Table 4 presents the standardized regression coefficients and Figures 1–3 presents a scatter diagram of the regression analysis. The *post hoc* test of the model was conducted based on the effect size (ƒ^2^) achieved. The effect size for the regression analysis in this study was ƒ^2^ = 0.33 (G*power 3.1), which is a medium-large effect according to Cohen’s (1988) guidelines. With 26 participants, 1 tested predictor, and an observed effect size of 0.33, the regression model had a power of 0.8. Despite the relatively small sample size, there is still good sensitivity to Type II errors.

## 4 Discussion

To the authors’ knowledge, this is the first study to explore the relationship between performance and sports visual ability in boxers. The results showed that of the ten visual ability variables, Visual Clarity, Contrast Sensitivity, Depth Perception, Perception Span, Multiple Object Tracking, Reaction Time, Eye-Hand Coordination, and Go/No Go have correlations, while there is no correlation with NFQ and TC. The main findings of this study showed that Reaction Time, Eye-Hand Coordination, and Depth Perception are important visual ability factors for boxers and can explain 92.5% of the variance in punch performance %Hit. We found varying levels of correlation between boxers’ punching performance and VC, CS, DP, PS, MOT, RT, EHC, and GNG, but not with NFQ and TC. This discrepancy may be due to the characteristics of different sports (Nascimento et al., 2020). The findings of this study are similar to those of Poltavski et al. and many studies that there is a correlation between visual ability variables and sports performance (Poltavski and Biberdorf, 2015) and that visual ability plays a key role in good sports performance in athletes (Blundell, 1985; Abernethy, 1986; Ciuffreda et al., 2004; Burris et al., 2018).

The mechanism behind the relationship between visual ability and sports performance can be explained by the Information Processing Model of Sports Performance (Welford, 1960; Erickson, 2018; Erickson, 2021). The model consists of three central processing mechanisms that operate sequentially: the perceptual mechanism, the decision mechanism, and the effector mechanism. The perceptual mechanisms are responsible for organizing and interpreting sensory information related to movement to facilitate optimal performance. Sensory information from various receptors (e.g., visual, vestibular, auditory, and tactile) is received during sports. Therefore the most relevant information needs to be selected for processing to perform the relevant task. Meanwhile, irrelevant sensory information will be filtered out. The sensory information that has been processed is communicated to the decision mechanism to determine the appropriate motor response strategy. In some situations, the decision mechanism may also inhibit motor responses, as sometimes it is appropriate not to react during a match. The effectiveness of decision processing can be influenced by the athlete’s sports knowledge and experience. Finally, the preferred motor response is transmitted from the decision mechanism to the effector mechanism. The effector mechanism organizes, initiates, and controls neural commands generated by appropriate brain centers for the performance of the selected motor response at the ideal time. Throughout the process, the perceptual mechanism and the decision mechanism continuously process internal and external sensory information to modulate the motor response when sufficient time exists to modify the response (Welford, 1960; Erickson, 2018; Erickson, 2021).

Sports vision has been used in many sports and the relationship between athlete performance and visual ability in these sports has been confirmed by many studies (Ludeke and Ferreira, 2004; Reichow et al., 2011; Mangine et al., 2014; Poltavski and Biberdorf, 2015; Burris et al., 2018; Laby et al., 2018; Laby et al., 2019; Liu et al., 2020; Laby and Appelbaum, 2021). These studies likewise support the findings in this study. Reaction time and response time (movement time) are considered the classic measures of how efficiently and effectively a person masters sports skills (Huppert et al., 2021). In this study, RT was the most important visual ability variable for boxers. This is similar to the findings of Poltavski and Biberdorf, (2015). on visual ability measures predicting actual game performance in hockey players. As found by Stanley et al., (2018); Menzel and Potthast, 2021 experienced boxers can throw punches in 402–405 ms, and in such a short time the boxer needs to react and duck quickly. The findings of Darby et al., (2014) similarly support the results of this study, suggesting that there is a correlation between reaction time and performance in boxing Mangine et al., (2014) reported no relationship between reaction time and performance metrics of NBA basketball players which is different from this study. Reaction Time is particularly important given the high velocity and short duration of attacking movements in combat sports like boxing (VencesBrito et al., 2011; Martínez de Quel and Bennett, 2019). Several other studies similarly support the results in this study regarding Reaction Time ability in boxers (Bianco et al., 2008; Laby et al., 2011; Badau et al., 2018). Although the measurements in these studies are different from those in this study, all agree that the Reaction Time of the boxer is optimal and that it is important for the boxer.

In the findings of the present study, EHC is another important visual ability variable in boxers. The skill of coordinating eye and body movements is known as eye-hand visual-motor reaction time, which is defined as the time interval between the appearance of a visual stimulus and the completion of a motor response with a limb (Erickson, 2022). Other scholars have described Eye-Hand Coordination as a series of decisions and resulting motor actions to accomplish a specific task (Boff et al., 1986; Ellison, 2015). This represents the integration of visual information, decision-making, and motor actions to accomplish a specific task at a rate that depends on visual, perceptual, and motor factors (Laby et al., 2018). The primary goal of a boxer in a match is to successfully throw a clean punch at an opponent without being returned (Guidetti et al., 2002), to observe the opponent through the eyes, to receive visual information about the opponent’s dynamics and react quickly, and to perform a large number of offensive and defensive maneuvers (Davis et al., 2013; Slimani et al., 2017). From this point of view, the visual-motor process of the boxer is consistent with the meaning of Eye-Hand Coordination. This is a good explanation of why EHC is an important element of visual ability for boxers in this study. The same can also explain the positive correlation between GNG and boxers’ punching performance in this study. In addition, the measurements in EHC and GNG consisted of rapidly touching or not touching a bright spot on the screen with either hand, depending on the target position. This approach appears to be capable of simulating eye-hand coordination tasks in boxing. Laby et al., (2018) reported similar results when they confirmed the role and relationship of assessing eye-hand coordination on baseball batting performance.

The vast majority of situations in sports require athletes to make specific and appropriate motor responses to specific visual stimuli (Poltavski and Biberdorf, 2015). Erickson et al. argue that the speed and accuracy with which vision is linked to neuromuscular processing is evidence of the integrity of the visuomotor control system (Erickson, 2022). In the present study, RT and EHC equally represent the ability of athletes to quickly recognize and accurately respond to visual stimuli. These two variables are important factors in explaining the punch accuracy of boxers.

In this study, Depth Perception was the third important variable of visual ability in boxers. Depth Perception reflects an athlete’s ability to quickly and accurately determine the distance of objects in front of them as well as their spatial positional relationships (Elmurr, 2011). Judgment of depth aids in navigation, accurate timing, and correct prediction of potential collisions (Erickson et al., 2011; Erickson, 2021). This is important for boxers. Because the boxer must not only accurately judge their opponent’s spatial position and distance, but also see the state of his opponent’s punches clearly to land accurate blows on them (Davis et al., 2013; Slimani et al., 2017; Davis et al., 2018; Martínez de Quel and Bennett, 2019). This is equally good in explaining the need for Depth Perception ability in boxers.

Among the other visual ability variables, the boxer’s %Hit was strongly correlated with VC, CS, and PS, whereas it was only moderately correlated with MOT and uncorrelated with NFQ and TC. Unlike in this study, did not find an association between static visual measures such as Contrast Sensitivity and Depth Perception and hockey players’ on-field performance, while dynamic visual measures including near-far quickness had a significant association (Poltavski and Biberdorf, 2015). Looking at the characteristics of hockey and boxing, hockey players compete in a much larger field, while boxers only play in a field of about 16*18 feet. In addition, hockey players need to focus on information about the dynamics of the ball and the players, who move randomly near and far. While boxers only need to focus on the positional information of their opponents and referees in the near distance. Thus, the ability to quickly switch focus near-far, the ability to target capture, and the ability to track multiple targets do not seem to be important to boxers. However, the moderate correlation between the MOT measure and the boxer’s punching performance in this study was unexpected. Boxers need to be aware of their opponent’s upper torso and chin area during a bout, as well as the peripheral movements of the gloves and body, in addition to always knowing where they are in the ring relative to the ropes, their opponent, and the referee. Thus, the ability to perceive span well seems to be helpful to boxers. Laby et al. also reported a correlation between visual clarity and contrast sensitivity and batting performance in professional baseball (Laby et al., 2019). The results of this present study were similar. However, the literature on the relationship between boxers’ punching performance and visual ability variables such as visual clarity and contrast sensitivity is still limited, as these results are preliminary.

Poltavski and Biberdorf, (2015) investigated the role of visual ability measures in predicting actual game performance in hockey players. They showed that 69% of the difference in forward goals could be predicted by better Reaction Time, Visual Memory Ability, Visual Discrimination Ability, and Ability to Switch Focus Between Near and Far Objects, which included Reaction Time, Go/No Go, Perception Span, and Near Far Quickness. This is similar to the results of this study. However, the difference is that this study is an exploration of the relationship between performance and visual ability in boxers. It is therefore characterized by the sport of boxing. Henrique et al. gave a good explanation for this by stating that athletes need good visual abilities to clearly acquire information in sports, but depending on the sport, certain abilities need to be more developed than others (Nascimento et al., 2020).

## 5 Limitations

The current study had some limitations. First, a natural limitation of conducting a study on elite athletes is the very limited number of participants. There were a limited number of elite boxers included in this study because only a few athletes from a single region met the study’s inclusion criteria. Thus some other potential relationships in the study may have been overlooked. Second, different sports have different requirements for athletes. The participants in this study were elite amateur boxers. Therefore, the application of the results of this study to other sports may be limited. In addition, another major limitation of this study is that it did not include a control group. Therefore it was not possible to compare the differences with other sports.

## 6 Conclusion

The results of this study demonstrated for the first time a relationship between sports visual ability and boxers’ punching performance. Among the visual ability variables, Visual Clarity, Contrast Sensitivity, Depth Perception, Perception Span, Multiple Object Tracking, Reaction Time, Eye-Hand Coordination, and Go/No Go are correlated with boxers’ punching accuracy, and the level of these visual abilities affects boxers’ in-match punching performance. In addition, Reaction Time, Eye-Hand Coordination, and Depth Perception are important visual ability variables for boxers, and their improved levels can lead to improved boxer punching performance. Boxers need to have faster reaction times, better processing of visual information and decision-making abilities, and the ability to accurately recognize the distance and position of their opponents and their punches. Therefore, the importance of Reaction Time, Eye-Hand Coordination, and Depth Perception for the boxer should be emphasized in actual boxing training, as well as the role of other visual abilities in boxing performance. It is recommended that visual ability assessment and sports vision training should be incorporated into actual boxing training. The characteristics of the sport of boxing should also be taken into account to design methods of visual ability training suitable for boxers to maximize boxing performance. Future research needs to explore the relationship between visual ability and boxers’ defensive performance, as well as the effects of visual training to enhance boxers’ on-field performance. In addition, future research needs to further select variables that more fully characterize boxers’ performance and confirm these results through more sophisticated analytical methods.

## Data Availability

The original contributions presented in the study are included in the article/Supplementary Material, further inquiries can be directed to the corresponding author.
